# Expression of angiopoietin-like 4 fibrinogen-like domain (cANGPTL4) increases risk of brain metastases in women with breast cancer

**DOI:** 10.18632/oncotarget.27553

**Published:** 2020-05-05

**Authors:** Tu Dao, Guillaume Gapihan, Christophe Leboeuf, Diaddin Hamdan, Jean-Paul Feugeas, Hanene Boudabous, Laurent Zelek, Catherine Miquel, Thuan Tran, Catherine Monnot, Stéphane Germain, Anne Janin, Guilhem Bousquet

**Affiliations:** ^1^Université Paris Diderot, Inserm, UMR_S942, Paris, France; ^2^Medical Oncology Department, National Cancer Hospital, Ha Noi, Vietnam; ^3^Ha Noi Medical University, Oncology Department, Ha Noi, Vietnam; ^4^INSERM, U722-Paris, Paris, France; ^5^Oncology Department, Hôpital Avicenne, APHP, Bobigny, France; ^6^Université Paris 13, Villetaneuse, Paris, France; ^7^Pathology Department, Hôpital St Louis, APHP, Paris, France; ^8^Center for Interdisciplinary Research in Biology (CIRB), College de France, CNRS, INSERM, PSL Research University, Paris, France; ^9^Cancer Research and Clinical Trial Center, National Cancer Hospital, Ha Noi, Vietnam; ^10^Université de Franche Comté, Besançon, France; ^*^These authors contributed equally to this work; ^#^These authors are co-senior authors

**Keywords:** cANGPTL4, brain metastases, breast cancer, blood-brain barrier, vascular permeability

## Abstract

Background: Brain metastases challenge daily clinical practice, and the mechanisms by which cancer cells cross the blood-brain barrier remain largely undeciphered. Angiopoietin-like 4 (ANGPTL4) proteolytic fragments have controversial biological effects on endothelium permeability. Here, we studied the link between ANGPTL4 and the risk of brain metastasis in cancer patients.

Materials and Methods: From June 2015 to June 2016, serum samples from 113 cancer patients were prospectively collected, and ANGPTL4 concentrations were assessed. Using a murine model of brain metastases, we investigated the roles of nANGPTL4 and cANGPTL4, the two cleaved fragments of ANGPTL4, in the occurrence of brain metastases.

Results: An ANGPTL4 serum concentration over 0.1 ng/mL was associated with decreased overall-survival. Multivariate analyses found that only breast cancer brain metastases were significantly associated with elevated ANGPTL4 serum concentrations.

4T1 murine breast cancer cells were transfected with either *nANGPTL4-* or *cANGPTL4*-encoding cDNAs. Compared to mice injected with wild-type 4T1 cells, mice injected with nANGPTL4 cells had shorter median survival (*p* < 0.05), while mice injected with cANGPTL4 had longer survival (*p* < 0.01). On tissue sections, compared to wild-type mice, mice injected with nANGPTL4 cells had significantly larger surface areas of lung metastases (*p* < 0.01), and mice injected with cANGPTL4 had significantly larger surface areas of brain metastases (*p* < 0.01).

Conclusions: In this study, we showed that a higher expression of Angiopoietin-like 4 Fibrinogen-Like Domain (cANGPTL4) was associated with an increased risk of brain metastases in women with breast cancer.

## INTRODUCTION

Breast cancer is a leading cause of cancer death in women [1]. Ten to 35% of patients with breast cancer will develop metastases with a median survival of up to 34.4 months [2]. Brain metastases occur in the progression of metastatic breast cancer in 15 to 40% cases [3], with a median survival of less than 15 months [4], challenging daily practice in oncology. In addition, the incidence of brain metastases has increased in the last ten years, as a result of better control of localizations outside the central nervous system, and because most chemotherapeutic agents fail to cross the blood-brain barrier [5].

The blood-brain barrier (BBB) is made up of endothelial cells separated by tight junctions that prevent paracellular diffusion of xenobiotics and cells from blood to brain [6–8]. The mechanisms by which cancer cells permeabilize and cross the BBB to form brain metastases remain to be deciphered.

Angiopoietin-like 4 (ANGPTL4) is a matricellular protein, initially described as an angiogenic factor in settings of vascular injury or tumor development [9–17]. The native full-length protein (FLANGPLT4), composed of 406 amino acids, is proteolytically cleaved into two fragments, a N-terminal coiled-coil domain (nANGPTL4) encompassing amino-acid 1 to 170, and an ANG/fibrinogen-like C-terminal domain (cANGPTL4) encompassing amino-acid 171 to 406 (Supplementary Figure 1). This proteolytic cleavage of FLANGPTL4 at the -RRXR- cleavage site by proprotein convertases depends on the tissue in which ANGPTL4 is synthesized and on the physiological or pathological circumstances [18–23].

*In situ* ANGPTL4 expression in tumor cells of primary tumors is associated with poor prognosis and metastatic relapse in patients with localized breast [14, 24–27], gastric [15, 28], head and neck [29], colon [30], and hepatocellular carcinoma [31–33]. In a preclinical murine model of brain metastases using human breast cancer cell lines, ANGPTL4 mRNA and protein expression were associated with an increased risk of lung and brain metastases [14, 24].

In preclinical studies, ANGPTL4 has been described both as a protector of the lung endothelium [12, 34], but also as a factor that increases vascular permeability [29], and potentially the risk of brain metastases [35, 36]. These context-dependent effects on endothelial cells could be linked to the different fragments, N- or C-terminal of the ANGPTL4 protein that may be present in tissues [17].

In the serum of 113 patients with different types of cancers, we initially assessed ANGPTL4 concentrations and showed that ANGPTL4 was elevated in women with breast cancer brain metastases. Using an experimental murine model of breast cancer brain metastases, we investigated the role of each cleaved fragment of ANGPTL4 in the occurrence of brain metastases.

## RESULTS

### ANGPTL4 serum concentration is associated with brain metastasis and decreased overall survival in breast cancer patients

Patient characteristics are detailed in Supplementary Table 1. Briefly, cancer types were breast (*n* = 38), lung (*n* = 44), prostate (*n* = 14), ovarian (*n* = 7), and others (*n* = 10). Patients had metastatic disease in 81.4% of cases, including bone (33.6%), lung (26%), liver (22.1%), and brain (20.3%) metastases.

**Figure 1:**
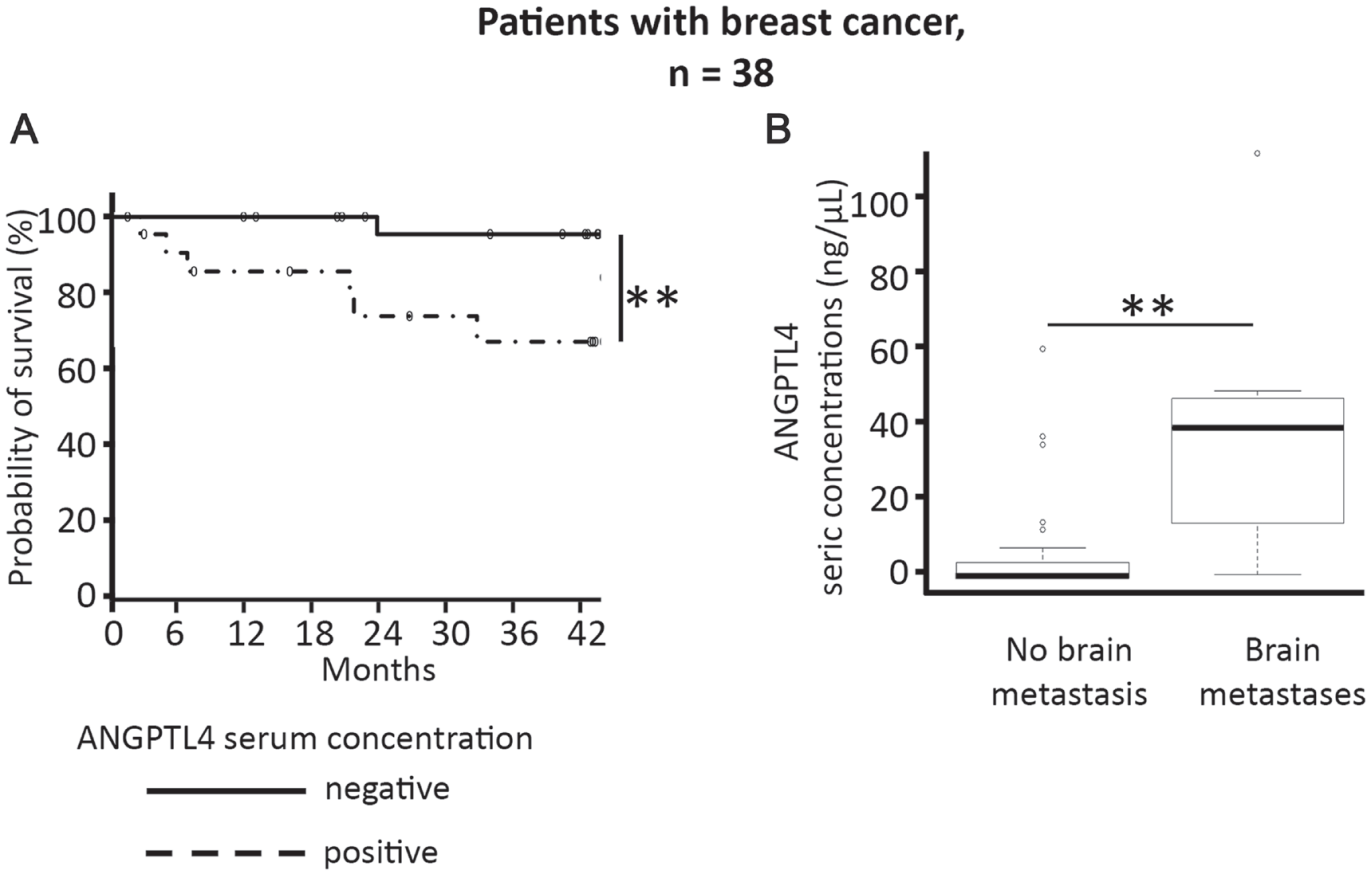
Elevated ANGPTL4 serum concentration is associated with shorter survival in the 38 women with breast cancer (**A**), and predicts the risk of brain metastases in these patients (**B**). ^*^ *p* < 0.05, ^**^ *p* < 0.01.

For the 38 women with breast cancer (Table 1), the mean age was 53.6 years. Twenty-one women (55.3%) had metastatic disease, including bone (34.2%), lung (31.6%), liver (23.7%), and brain (21.0%) metastases. In this sub-group of 38 women with breast cancer, the mean ANGPTL4 serum concentration was 5.6 ng/mL, ranging from 0 to 52.8 ng/mL. Positive ANGPTL4 serum concentration was significantly associated with overall survival (55.5% patients alive at 42 months versus 93.8%, respectively, *p* < 0.01, Figure 1A).

**Table 1:** Characteristics of breast cancer patients

Parameter	*N* (38)	%
**Age, years (mean, range)**	53.6 (38–93)	
**Histological subtype**		
HER2 3+	12	31.6
TNBC	9	23.7
ER+, HER2–	6	15.8
ER+, PR+, HER2–	6	15.8
HER2 3+, ER+, PR–	4	10.5
HER2 3+, ER+, PR+	1	2.6
**Localized stage**	17	44.7
**Metastatic stage**	21	55.3
**Brain/Leptomeningeal metastases**	8	21.0
At the time of ANGPTL4^*^ testing		
** Non-progressive disease**	18	47.4
** Progressive disease**	20	53.6
**Total**	**38**	**100**

HER2: human epidermal growth factor receptor 2, TNBC: triple negative breast cancer, Localized stage: local or loco-régional disease including lymph-node metastases; Metastatic stage: distant metastases, ER: estrogen receptor, PR: progesterone receptor, ^*^angiopoietin like-4.

By contrast, in all 112 cancer patients, with a mean ANGPTL4 serum concentration of 4.9 ng/mL, ranging from 0 to 107.8 ng/mL, a positive ANGPTL4 serum concentration was not significantly associated with shorter overall survival.

Multivariate analyses according to type of cancer and metastatic localization found that only breast cancer brain metastases were significantly associated with elevated ANGPTL4 serum concentration (*p* < 0.05) (Figure 1B, and Supplementary Figure 2). For breast cancer patients, a significant association was systematically found between positivity of ANGPTL4 serum concentration and brain metastasis, whatever the level of positivity for ANGPTL4 serum concentration (*p* < 0.01, Supplementary Figure 3). Using a cut-off for positivity of over 0.1, the sensitivity for association with brain metastases was 100% and the specificity 63%.

In summary, we found that positive ANGPTL4 serum concentration over 0.1 ng/mL was associated with an increased risk of brain metastases and shorter survival in patients with breast cancer.

### ANGPTL4 fragments were differently expressed in tumor cells of breast cancer brain metastases

We assessed cANGPTL4 and nANGPTL4 expression in tumor cells of brain metastases (*n* = 28), lung metastases (*n* = 10), and liver metastases (*n* = 10) from breast cancer patients.

**Figure 2:**
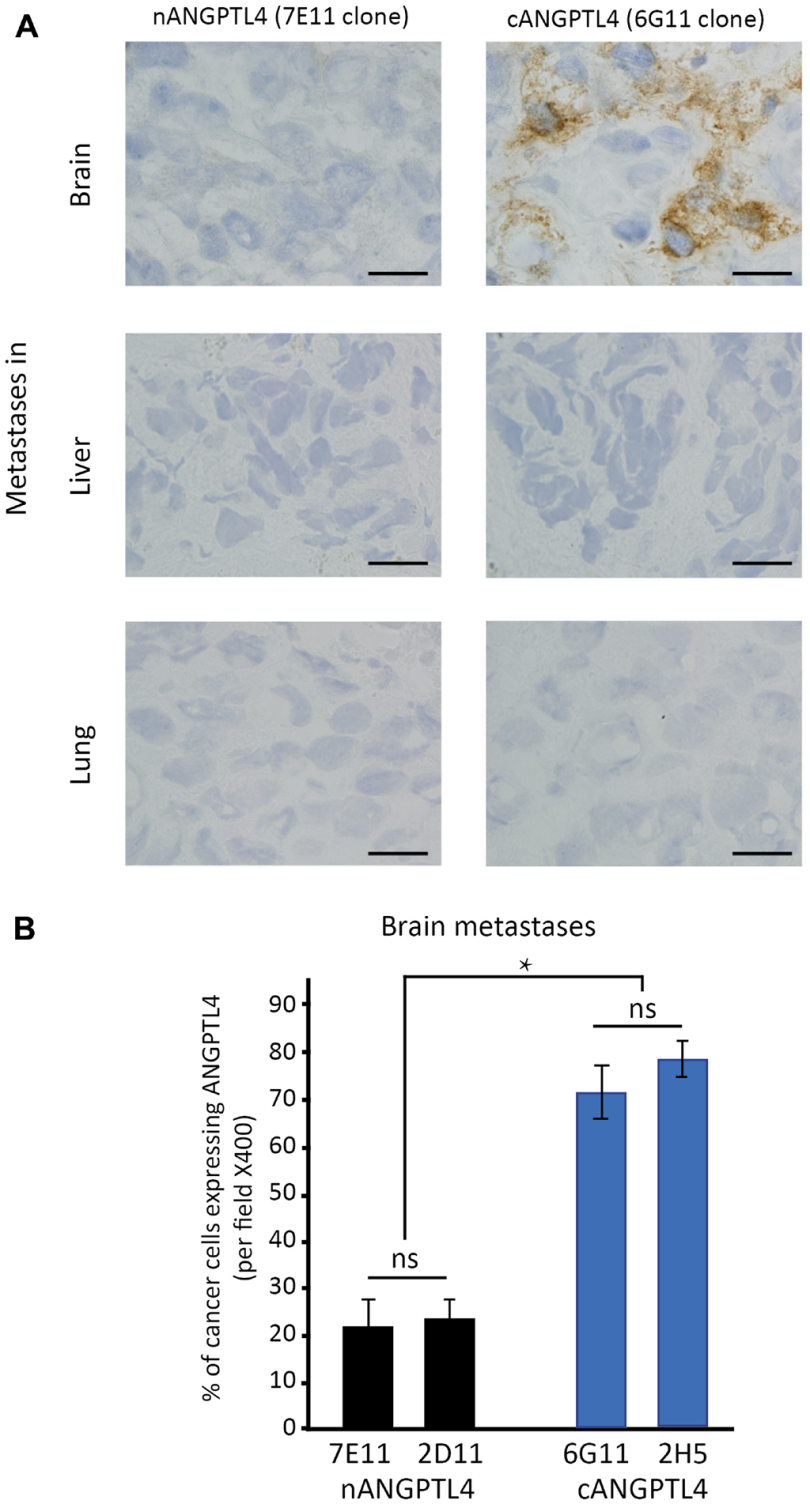
Differential expression of ANGPTL4 fragments in brain metastases of women with breast cancer. (**A**) shows immunostainings for cANGPTL4 and nANGPTL4 fragments in brain, lung and liver metastases of women with metastatic breast cancer. cANGPTL4 is significantly more expressed in cancer cells of brain metastases than nANGPTL4 (**B**). 7E11 and 2D11 are antibodies recognizing nANGPTL4 fragment, and 6G11 and 2H5 are antibodies recognizing cANGPTL4 fragment. ^*^ *p* < 0.05, ns: not significant. Bar scale: ×400.

In brain metastases, we found a significantly larger number of tumor cells expressing the cANGPTL4 fragment than cells expressing the nANGPTL4 fragment (75% vs. 22%, respectively, *p* < 0.01, Figure 2).

For antibodies recognizing the same fragment, there was no difference between the two antibodies.

In liver metastases, there was no expression of the nANGPTL4 fragment or the cANGPTL4 fragment. In lung metastases, only one case was positive for the two ANGPTL4 fragments.

### ANGPTL4 fragments induce different cell effects *in vitro*

Since ANGPTL4 is cleaved into nANGPTL4 and cANGPTL4 fragments [19], we aimed to decipher the biological effect of each of the two fragments when expressed by 4T1 murine breast cancer cells.

**Figure 3:**
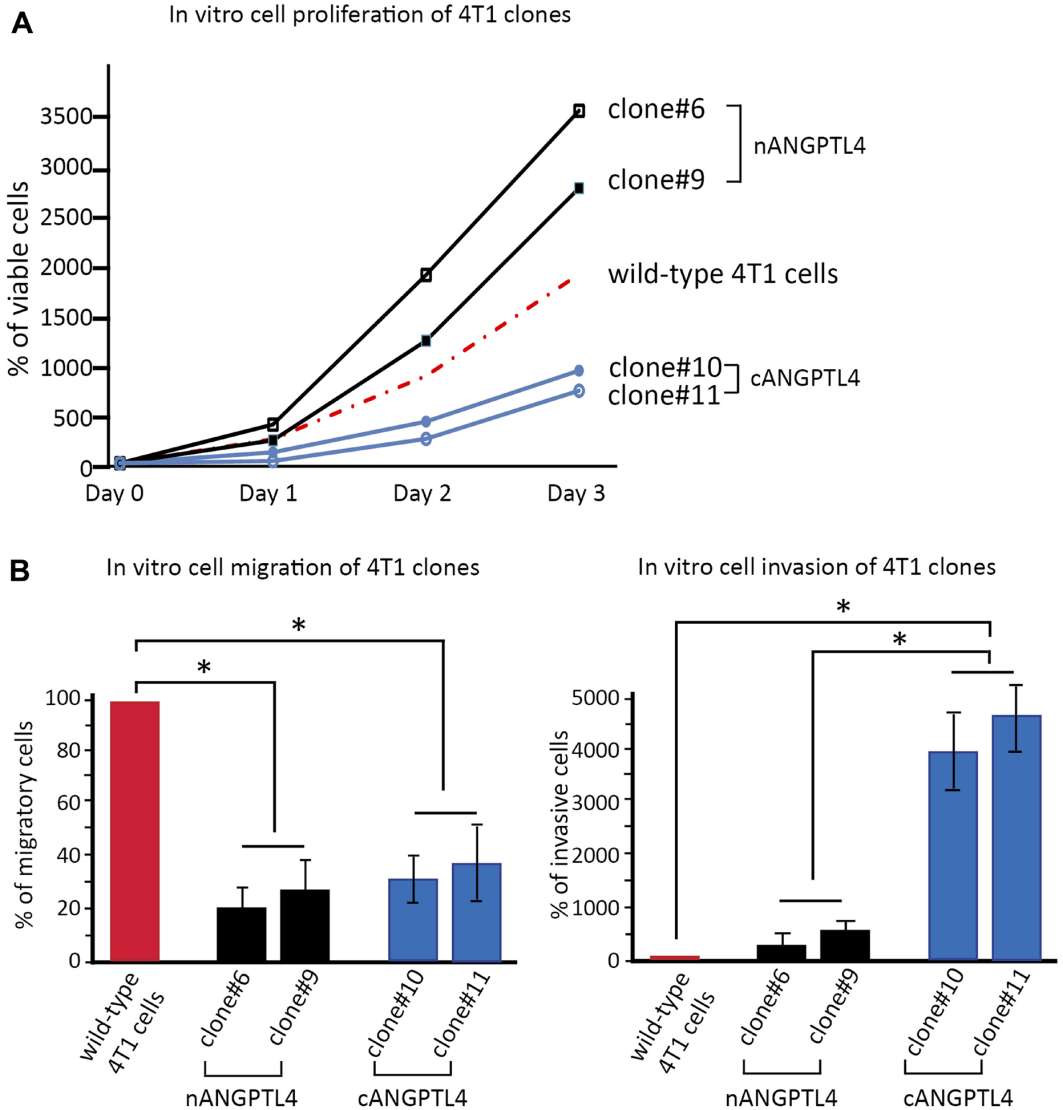
*in vitro* biological effects of c- and nANGPTL4 fragments on 4T1 murine breast cancer cells. (**A**) 4T1 cells expressing nANGPTL4 (clones #6 and #9) exhibit greater proliferation capacities than wild-type cells or 4T1 cells expressing cANGPTL4 (clones #10 and #11). (**B**) nANGPTL4 and cANGPTL4 cells have a lower migration potential than wild-type 4T1 cells (left panel), and cANGPTL4 cells exhibit significantly greater invasiveness than nANGPTL4 and wild-type cells (right panel). ^*^ *p* < 0.05, ^**^ *p* < 0.01.

We first assessed cell proliferation on the different clones transfected with either nANGPTL4 or cANGPTL4 fragments. We found that 4T1 cells transfected with nANGPTL4 (clones #6 and #9) had a higher proliferation rate at Day 3 than 4T1 wild-type cells (*p* < 0.05, Figure 3A). Conversely, 4T1 cells transfected with cANGPTL4 (clones #10 and #11) had a significantly lower proliferation rate than 4T1 wild-type cells (*p* < 0.05, Figure 3A).

Cell migration was significantly lower for 4T1 cells expressing cANGPTL4 and for 4T1 cells expressing nANGPTL4 than for 4T1 wild-type cells (*p* < 0.01, Figure 3B, left panel).

Cell invasion was significantly higher with cANGPTL4 4T1 cells compared to 4T1 wild-type cells (*p* < 0.01, Figure 3B, right panel).

For all experiments (proliferation, migration, invasion), there was no difference between the two clones expressing nANGPTL4, nor between the two clones expressing cANGPTL4.

### Increased brain metastases with 4T1 breast cancer cells expressing cANGPTL4

For *in vivo* experiments, 4T1 wild-type cells and the four different clones expressing either nANGPTL4 or cANGPTL4 were injected intravenously into syngeneic mice.

**Figure 4:**
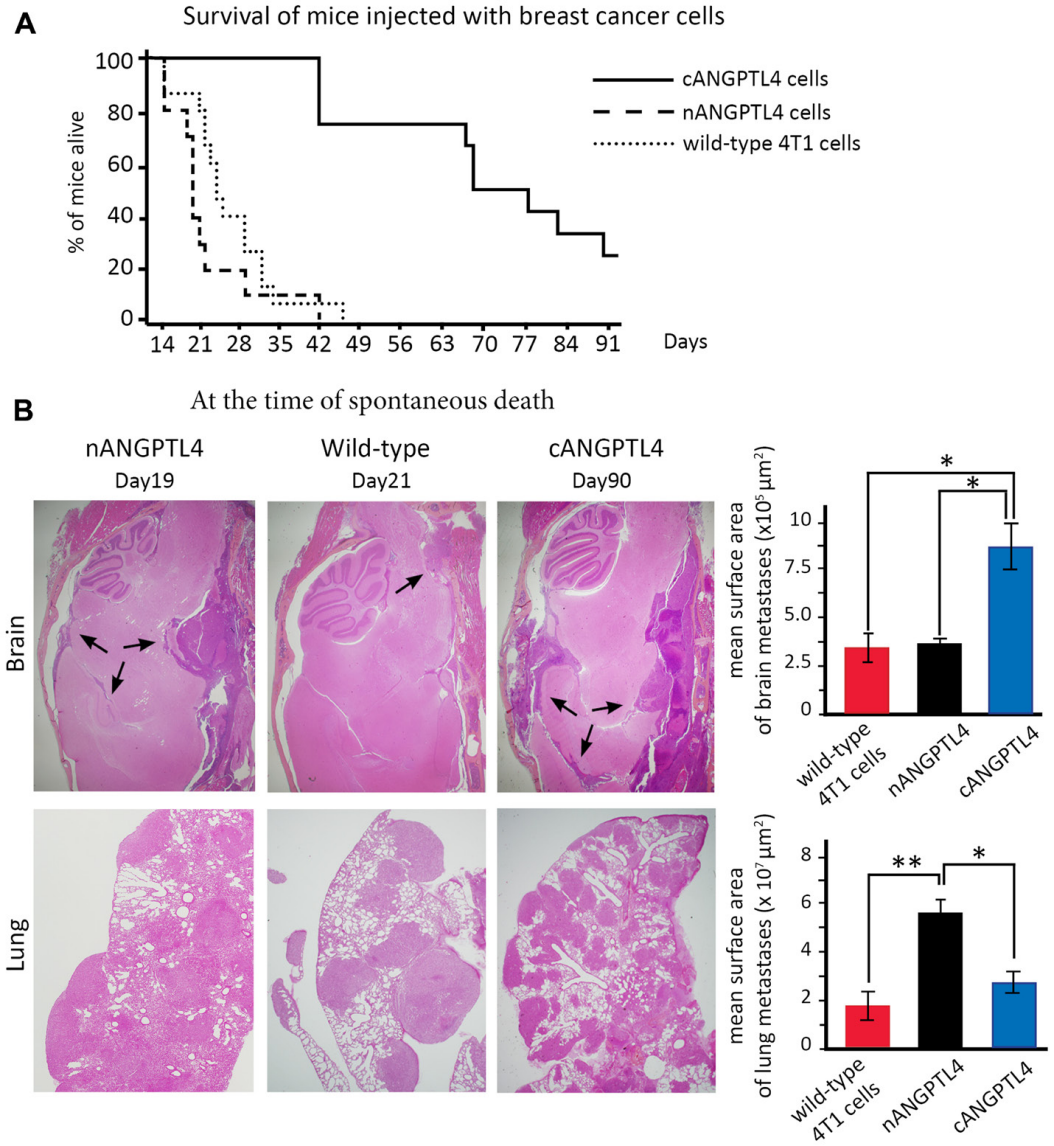
Phenotype of mice injected with 4T1 cells expressing either nANGPTL4 or cANGPTL4 fragment. (**A**) Kaplan-Meier curves for overall mouse survival. Survival is significantly increased for mice injected with cANGPTL4 cells compared to mice injected with wild-type or nANGPTL4 cells. (**B**) Haematoxylin-eosin stained section of brain and lung metastases obtained at the time of spontaneous death for three mice injected with either wild-type, or nANGPTL4, or cANGPTL4 4T1 cells. At this date, the surface area of brain and meningeal metastases (black arrows) is significantly larger for mice injected with cANGPTL4 cells than for other mice. Lung and pleural metastases are numerous in all three types, with a considerable and significantly larger metastatic burden for nANGPTL4 cells. ^*^ *p* < 0.05, ^**^ *p* < 0.01. ns: not significant.

Mice injected with nANGPTL4-expressing cells had a shorter median survival of 19 days, while death occurred after a median time of 23 days for mice injected with 4T1 wild-type cells. In contrast, median survival was largely increased (66 days) for mice injected with cANGPTL4-expressing cells (Figure 4A).

At the time of spontaneous death (Figure 4B), histological analyses showed that mice injected with nANGPTL4 cells died from massive pleural and lung metastases (mean surface area of 5.5 × 10^7^ μm^2^, lower panel), and that they had brain metastases with a mean surface are of 3.2 × 10^5^ μm^2^ (upper panel). For mice injected with wild-type 4T1 cells, lung and pleural involvement was significantly less massive with a mean surface area of 1.9 × 10^7^ μm^2^ (*p* < 0.01, lower panel), while the surface area of brain metastases was comparable to that observed with nANGPTL4 cells. For mice injected with cANGPTL4 cells, the mean surface area of lung and pleural metastases was 2.8 × 10^7^ μm^2^ (lower panel), while the surface area of brain metastases was significantly larger (8.8 × 10^5^ μm^2^, *p* < 0.05, upper panel) than in mice injected by wild-type 4T1 cells or nANGPTL4 cells.

**Figure 5:**
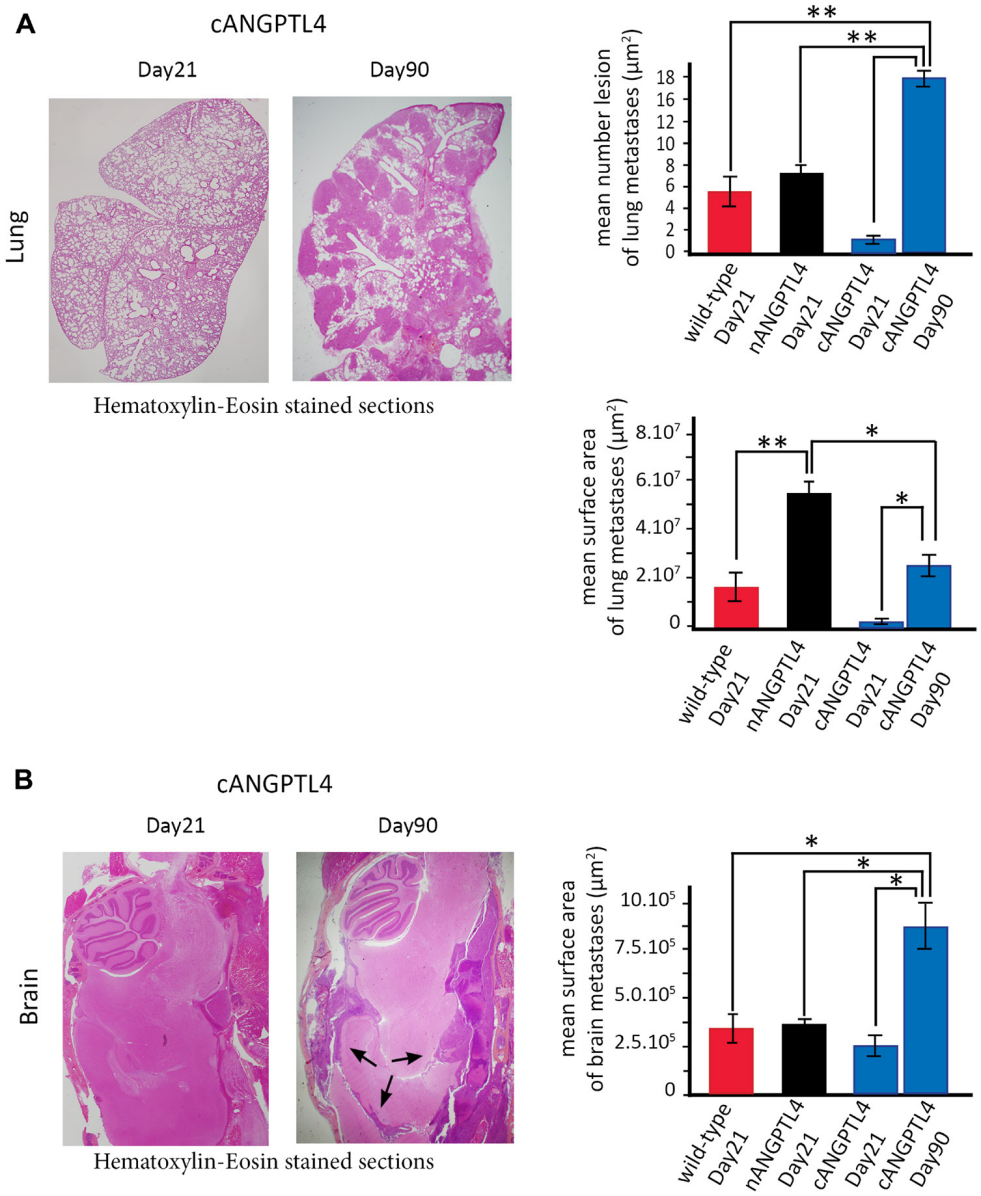
Mouse phenotype with cANGPTL4 4T1 cells at two different time-points, Day 21 and at the time of euthanasia. (**A**) Lung metastatic burden significantly increases between Day 21 and Day 90 in mice injected with cANGPTL4 cells. At Day 21, there are limited to small areas or vascular emboli (left panel). At Day 90, there are numerous but of smaller size than those observed at Day 21 in mice injected with wild-type and nANGPTL4 4T1 cells. (**B**) Brain metastatic burden significantly increases between Day 21 and Day 90 in mice injected with cANGPTL4 cells. At Day 90, the mean surface area of brain metastases (black arrows) is significantly larger than that observed at Day 21 in mice injected with wild-type and nANGPTL4 4T1 cells. ^*^ *p* < 0.05, ^**^ *p* < 0.01.

To compare mice with the same time of death, we injected a new series of 10 mice as above and euthanatized them at Day 21. In line with their increased median survival, we found limited lung or pleural involvement, with the presence of vascular tumor emboli in mice injected with cANGPTL4 cells (Figure 5A). In contrast, meningeal and brain metastases were already present, with a mean surface area of metastases comparable to mice injected with wild-type 4T1 or nANGPTL4 cells and also analyzed at Day 21 (Figure 5B).

Overall, mice injected with nANGPTL4 cells had the shortest survival as a result of massive lung and pleural involvement, while mice injected with cANGPTL4 cells had the longest survival with more extensive brain and meningeal metastases.

## DISCUSSION

In our study, we found that a positive serum concentration of ANGPTL4 over 0.1 ng/mL was associated with an increased risk of brain metastasis in women with breast cancer, and with shorter survival. Previous studies have reported that elevated ANGPTL4 serum concentrations were associated with high-grade tumors in women with localized breast cancer [37], and with hepatic metastatic risk in hepatocellular carcinoma [31]. However, the translational value of ANGPTL4 serum concentration assessment has not yet been deciphered. In women with metastatic breast cancer, this could be performed prospectively in clinical practice to determine the risk of brain metastases more efficiently. Systematic screening by magnetic resonance imaging of the brain is not recommended [38, 39], mainly because we are not able to accurately predict individual risk. However, early detection of occult brain metastases significantly decreases the risk of cerebral death (from 48% to 16%) [38, 39], and should improve patient quality of life. Such screening could be proposed to women with metastatic breast cancer presenting ANGPTL4 serum positivity.

We did not find such an association between ANGPTL4 serum positivity and brain metastases in other cancer types, particularly for lung cancer, suggesting this association may only be true for breast cancer. However, we did not test any patient with melanoma, another cancer type associated with a high risk of brain metastasis [40]. Further studies are warranted to see if our results are only valid for breast cancer.

To determine the respective roles of cANGPTL4 and nANGPTL4 in brain dissemination of breast cancer cells, we first assessed their expression in breast cancer brain metastases. Using immunostaining, we found a differential expression of the two fragments, with a predominance of the cANGPTL4 fragment. Experimentally, using 4T1 murine breast cancer cell lines expressing one fragment or the other, we demonstrated their differential effect in terms of metastasis distribution after intravenous injection into syngeneic mice, and a preferential brain tropism for cells expressing the cANGPTL4 fragment.

These different phenotypes could be explained by a differential biological effect of ANGPTL4 fragments on tumor cell proliferation and invasiveness. In particular, lung metastatic burden could be linked to cell proliferation, and the delayed lung involvement with cANGPTL4 4T1 cells could be related to their low proliferative capacities. On the other hand, this delay in the occurrence of visceral metastases, combined with increased invasiveness, could explain the preferential brain tropism of cANGPTL4 4T1 cells. In women with HER2-overexpressing metastatic breast cancer, brain metastases occur in 50% of cases with a median time-lapse of 13.1 months [3, 41] as a result of excellent control of localizations outside the central nervous system and failure of most chemotherapeutic agents to cross the blood-brain barrier.

The different phenotypes observed in mice could also be explained by a differential biological effect of circulating ANGPTL4 fragments on endothelial cells and endothelium permeability. Most preclinical studies have been performed using cancer cells transfected with full-length ANGPTL4 protein, with controversial results. ANGPTL4 enhanced trans-endothelial passage of breast and hepatic cell lines *in vitro,* and increased liver and lung metastatic risk [25, 27, 42]. Conversely, murine melanoma or lung cancer cells transfected with full-length ANGPTL4 and injected subcutaneously to syngeneic mice decreased lung and brain metastatic dissemination, suggesting a protective effect of ANGPTL4 on lung microvascular disruption [12, 35]. None of these studies were able to assess the respective roles of cANGPTL4 and nANGPTL4 on metastasis dissemination, particularly in the brain. The blood-brain barrier is composed of endothelial cells with tight, continuous junctions whose molecular composition is complex, including integral membrane proteins such as occludin, junctional adhesion molecules, and claudin. *In vitro*, cANGPTL4 interacts with integrin α5β1, VE-cadherin, and claudin-5 to disrupt endothelial cells and thus to increase vascular permeability [43]: this could be how cancer cells expressing and secreting cANGPTL4 could disrupt and thus cross the blood-brain barrier. In other pathological circumstances such as anthrax infection or ovarian cancer, pleural and peritoneal effusion is associated with endothelium disruption and down-regulation of claudin-5 expression [44, 45]. In our murine models, pleural metastatic involvement was constantly associated with the presence of brain/meningeal metastases.

In conclusion, our study showed that a higher expression of Angiopoietin-like 4 Fibrinogen-Like Domain (cANGPTL4) was associated with an increased risk of brain metastases in women with breast cancer. Our results open the way for further translational research on the role of the cANGPTAL4 fragment as a biomarker to predict the risk of breast cancer brain metastasis, and as a potential target for the prevention of these metastatic localizations.

## MATERIALS AND METHODS

### Patients and sampling

Two series of patients with cancers were studied: i) one series for serum sampling; and ii) one series for tissue analyses of brain, liver, and lung metastases.

For the first series, the serum of 113 patients with cancer was prospectively and randomly collected between June 2015 to June 2016, regardless of tumor type and disease stage. At the time of serum sampling, all patients had undergone whole-body computed tomography within the last month, including brain imaging.

For the second series, frozen samples from surgically removed breast cancer brain metastases were provided by Hôpital Sainte-Anne Tumorbank, and frozen biopsy samples of liver or lung metastases of breast cancers were provided by Hôpital Saint-Louis Tumorbank.

In compliance with French Bioethics law (2004-800; June 8, 2004), all patients had been informed of the research use of the part of their samples remaining after diagnosis had been established, and did not oppose it. Informed consent was obtained for each patient. The Clinical Research Board Ethics Committee (Comité de Protection des Personnes) approved this study (CPP Ile de-France #13218).

### Serum and ANGPTL4 concentrations

For each patient, a minimum volume of 1 mL blood was collected. After blood sampling, whole blood was centrifuged for 15 minutes at 800G, 400 μL of serum was collected and stored at –20°C.

ANGPTL4 serum concentration was assessed by a home-made sandwich ELISA assay [9, 10] using IgG monoclonal antibodies directed against human N-terminal and C-terminal ANGPTL4 fragments (Biotem, France). We used two mouse monoclonal antibodies recognizing the N-terminal epitope of human ANGPTL4 (2D11 and 7E11) and two antibodies recognizing the C-terminal epitope of human ANGPTL4 (2H5 and 6G11) (Supplementary Figure 1). Antibodies 2D11 (IgG2b) and 2H5 (IgG2b) were used as capture antibodies to coat the wells, and antibodies 7E11 (IgG2b) and 6G11 (IgG2b) were used as the detection antibodies. We used Human Angiopoietin-like 4 full length protein (Abcam, UK) as a positive control. The secondary antibody was an anti-mouse IgG1 antibody (Jackson, USA). Enzymatic reaction was detected by p-nitrophenyl phosphate (PNPP) substrate solution (Thermo Scientific, USA). ANGPTL4 concentration was obtained by reading sample optical densities at 405 nm–550 nm wavelength.

### Metastatic samples and ANGPTL4 expression

An indirect immunoperoxidase method using 7E11 or 6G11 as primary antibodies was performed on 5 μm-thick frozen tissue sections of each metastatic sample. The secondary antibody was a rabbit monoclonal anti-mouse IgG1 H&L (clone M1gG51-4, Abcam, UK) coupled with antirabbit OmniMap detection kit (Roche diagnostic, Meylan, France). The systematic controls used were absence of primary antibody and use of an irrelevant primary antibody of the same isotype.

For all tissue sections and for each antibody, cells expressing nANGPTL4 or cANGPTL4 were counted independently by two pathologists (AJ, CL) on five different fields at ×400 magnification. A ProvisAX70 microscope (Olympus, Tokyo) with wide-field eyepiece number 26.5 was used, providing a field size of 0.344 mm^2^.

For each field, 100 tumor cells were analyzed. The percentage of nANGPTL4 or cANGPTL4-expressing cells was the number of positive cells among these 100 tumor cells. Results were expressed as mean ± standard error of the mean (SEM).

We were well aware that part of the staining was linked to antibody recognition of FLANGPTL4. However, the difference in number of cells expressing cANGPTL4 or nANGPTL4 on sister following tissue sections was necessarily linked to cANGPTL4 or nANGPTL4 fragment.

### Murine breast cancer cell line and cell culture

4T1, a murine triple negative breast cancer cell line, was obtained from the ATCC (Rockville, MD, USA). We chose 4T1cells for their brain metastatic potential when injected in syngeneic mice [46]. Cells were grown as monolayers in RPMI-1640 medium supplemented with 10% fetal calf serum (Invitrogen, France), 2 mM L-glutamine, 100 units/mL penicillin and 100 μg/mL streptomycin (PAA, France). All cell cultures were split at least once a week using trypsin/EDTA 0.02% (Invitrogen, France). All cells were tested regularly for Mycoplasma contamination by PCR (Stratagene, USA).

### Transfection of 4T1 cells with nANGPTL4 and cANGPTL4 genes

Western-blot analysis did not show any expression of ANGPTL4 in native 4T1 cells.

Two Plasmids, pCDNA3.1- CCD_22-170_ -myc-his and pCDNA3.1-FLD _171-406_ -myc-his, were used for transfection of 4T1 cells with nANGPTL4 and cANGPTL4 genes respectively, as previously described [47].

Cells were transfected at 90% confluence in 6-well plates using 4 μg DNA and Lipofectamine 2000 transfection reagent (Invitrogen, France). Transfected cells were selected by their resistance to 120 μg/ml of G418 antibiotic (Life Technologies Inc, UK) and cloned by limiting dilution. Each clone was controlled for efficient transfection by western-blot analyses using anti-myc antibody (9E10) from Roche Diagnostics (Basel, Switzerland).

For each gene, two clones were used for further experiments: clones #6 and #9 for nANGPTL4, clones #10 an #11 for cANGPTL4.

### *In vitro* assessment of proliferation

Cell proliferation was assessed for wild type 4T1 cells and for the different cANGPTL4 and nANGPTL4 clones. On the first day, 3.10^4^ cells were seeded in a 96-well plate and incubated at room temperature. Cell viability was assessed every day for 3 days by the colorimetric conversion of tetrazolium MTT (3-[4, 5-dimethylthiazol-2-yl]-2,5-diphenyltetrazolium bromide; Sigma, France) into formazan, to estimate the relative number of viable cells [48]. After incubation, the supernatant was discarded, and the cell pellet re-suspended in 0.1 ml of DMSO. The absorbance was measured at 560 nm using a Fluostar Optima microplate reader (BMG LabTech, France). Each experiment was performed in triplicate.

### *In vitro* assessment of migration and invasion

Cell migration assessments were performed using Boyden chambers on 12-well plates, with cell culture insert of 10.3 mm diameter and membrane pores of 8 μm size (Becton Dickinson, France). For invasion assays, we used Boyden chambers coated with Matrigel (Falcon, MA, USA). Migration and invasion were assessed on 4T1 cells and for the different nANGPTL4 and cANGPTL4 clones. For each experiment, 3 × 10^4^ cells were seeded in the upper chamber and cultured in high glucose DMEM medium supplemented with 10% fetal bovine serum at 37°C in a 5% CO2 atmosphere.

After 24 hours incubation for migration, and 5 hours for invasion, cells on the lower face of the filter were fixed, stained with crystal violet (Sigma Aldrich, USA), and counted. Ten fields were analyzed on each filter, and results were expressed as mean ± standard error of the mean (SEM). Each procedure was performed in triplicate.

### Murine models

For *in vivo* experiments, six-week-old CB17 SCID mice (Janvier Lab, France), maintained in specific pathogen-free conditions were intravenously injected with 200 000 cells of each of the following types: i) wild-type 4T1 cells (*n* = 15); ii) 4T1 cells expressing nANGPTL4 (*n* = 10 for each of the two clones); and iii) 4T1 cells expressing cANPTL4 (*n* = 10 for each of the two clones). The University Institute Board Ethics Committee for experimental animal studies approved this study (#2012-15/728-0115). The mice were followed up, with daily observation of weight loss, grooming behaviors, posture, respiratory rate and activity. At the time of spontaneous death (mice showing signs of severe suffering) or at Day 21, mice were euthanized by cervical dislocation. All organs were formaldehyde-fixed and paraffin-embedded for further histological analyses, performed by two pathologists (AJ, GB) unaware of the clone injected and the time of death.

Assessment of metastatic extension in each organ was performed on three consecutive virtual slides created on a Nanozoomer 2.0 HT scanner (Hamamatsu, Japan) on hematoxylin-eosin stained tissue sections. Areas of metastases were delineated on the virtual slides and quantified using DotSlide software, and the mean surface area of lung or brain metastatic extension was calculated in each mouse. Then a mean surface of metastatic extension was calculated for all animals of each group (*n* = 15 mice for 4T1 wild-type cells, *n* = 20 mice for 4T1 cells expressing nANGPTL4, and *n* = 20 mice for 4T1 cells expressing cANGPTL4).

### Statistical analyses

The R program (R version 3.2.0 copyright © 2015 The R Foundation for statistical computing) was used for the statistical analyses.

Quantitative variables were expressed as means ± standard deviation (SD) and categorical variables as numbers and percentages.

Multivariate analysis of the factors associated with brain metastasis used Wilcoxon’s signal rank test or Anova test for quantitative variables, and Chi-squared test or Fisher’s exact test for categorical variables, as appropriate.

Overall survival was estimated for all patients and for breast cancer patients in relation to serum concentration of ANGPTL4, using Kaplan Meier analysis (*P*-value for log rank test). Mouse survival was estimated according to the type of ANGPTL4 fragment. A two-tailed *P*-value of 0.05 was required for statistical significance.

## SUPPLEMENTARY MATERIALS


