# Supplementary material for: Expression of angiopoietin-like 4 fibrinogen-like domain (cANGPTL4) increases risk of brain metastases in women with breast cancer
Source: Oncotarget. 2020 May 5;11(18):1590–602. doi: 10.18632/oncotarget.27553 (PMC7210011; doi:10.18632/oncotarget.27553)
Supplement: Supplementary file 1 [file oncotarget-11-1590-s001.pdf]

Expression of angiopoietin-like 4 fibrinogen-like domain (cANGPTL4) increases risk of brain metastases in women with breast cancer

SUPPLEMENTARY MATERIALS

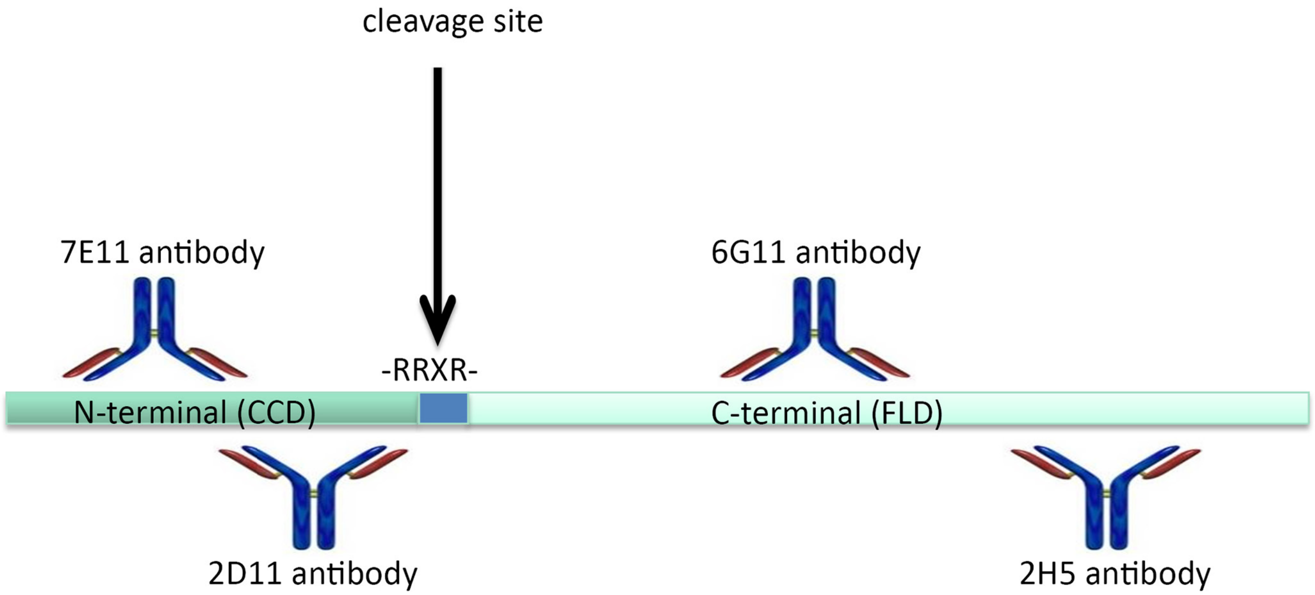

Supplementary Figure 1: Diagrammatic representation of ANGPTL4 protein. The ANGPTL4 protein is composed of a N-terminal (CCD) and a C-terminal domain (FLD). CCD: coiled-coil domain, FLD: ANG/fibrinogen-like COOH 7E11 and 2D11 are two monoclonal antibodies designed to recognize the nANGPTL4 epitope. 6G11 and 2H5 are two monoclonal antibodies designed to recognize the cANGPTL4 epitope.

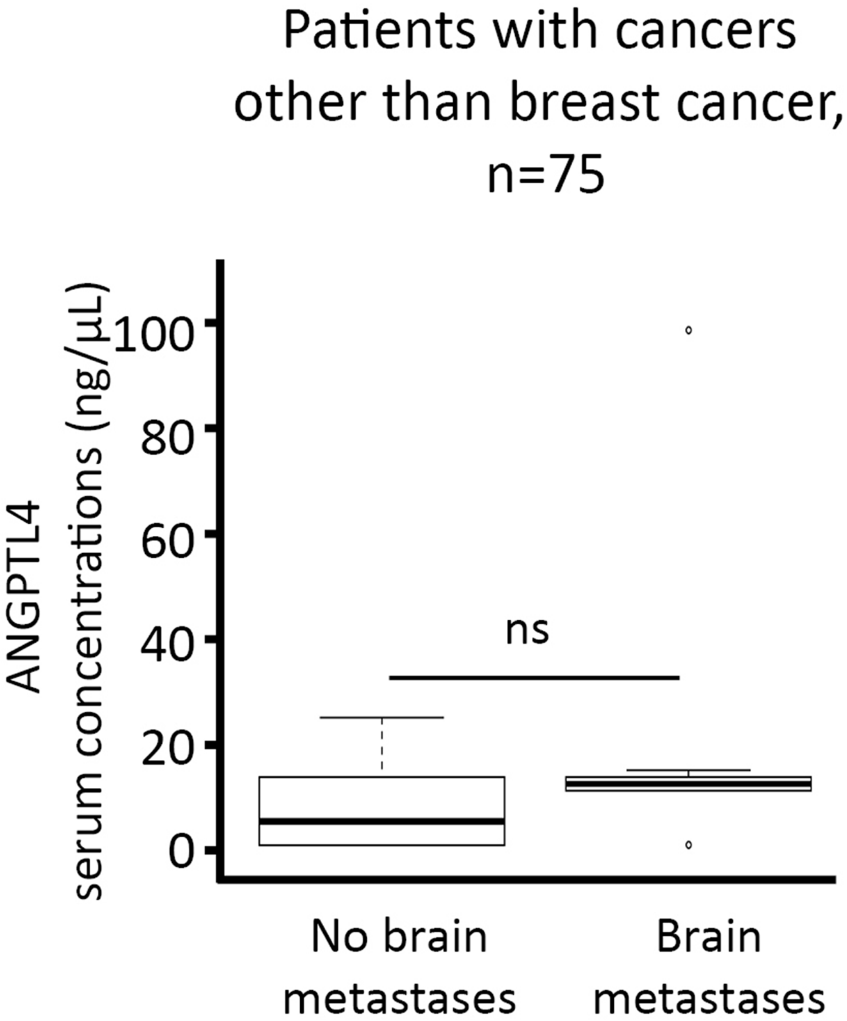

Supplementary Figure 2: ANGPTL4 serum concentrations in 75 patients with cancer others than breast cancer. No difference is observed according to the presence or absence of brain metastases. Abbreviation: ns, not significant.

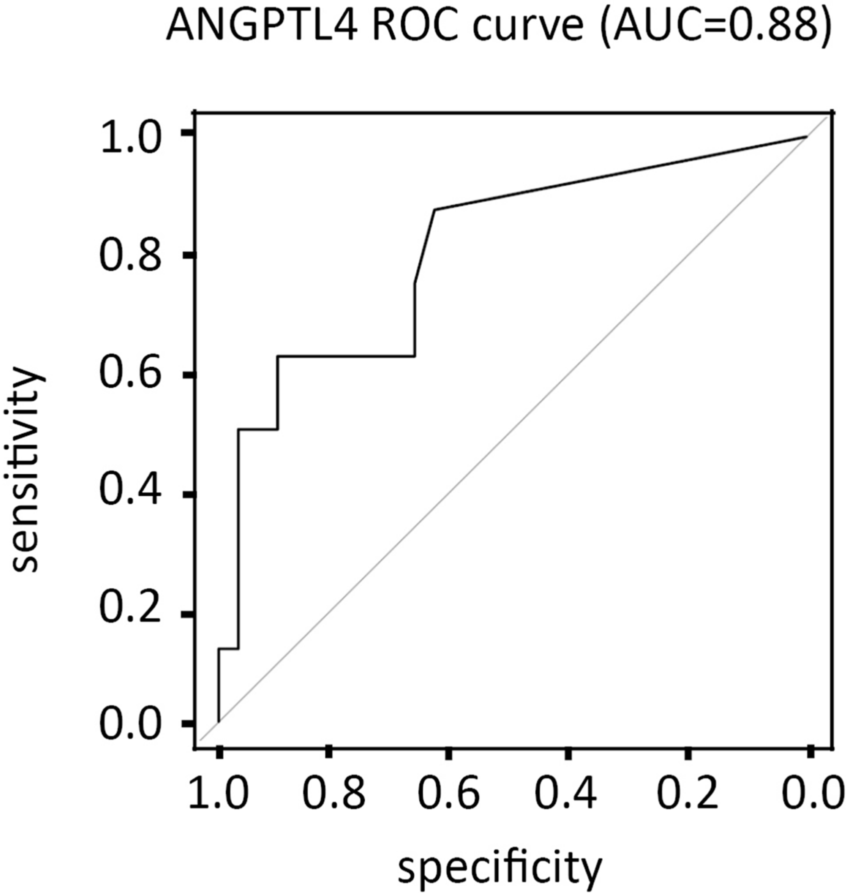

Supplementary Figure 3: Discrimination of ANGPTL4 serum concentrations between women with and without brain metastases. ROC curve discriminates breast cancer patients with and without brain metastases, according to ANGPTL4 serum concentration. Abbreviations: ROC, receiver operating characteristic; AUC, area under the curve.

Supplementary Table 1: Characteristics of the 113 patients

	N	%
**Age, years** (mean, range)	58.5<br>(35–93)	
**Primary cancer**		
Breast	38	33.7
Lung	44	38.9
Prostate	14	12.4
Ovarian	7	6.2
Colorectal	3	2.6
Kidney	2	1.7
Others	5	4.5
**Localized stage**	21	18.6
**Metastatic stage**	92	81.4
Brain metastases	25	20.3
Lung metastases	32	26.0
Liver metastases	25	22.1
Bone metastases	38	33.6
Lymph node metastases	35	26.5
Other metastases	20	17.7
**Mean ANGPTL4 concentration**<br>(range)	4.89 ± 7.11<br>(0–107.8)	

Abbreviation: SD, Standard deviation.
